# Supplementary material for: Rapid turnover of life-cycle-related genes in the brown algae
Source: Genome Biol. 2019 Feb 14;20:35. doi: 10.1186/s13059-019-1630-6 (PMC6374913; doi:10.1186/s13059-019-1630-6)
Supplement: Supplementary file 3 — Protein alignments, corresponding Gblocks html file and CDS sequences for the lineage conserved gametophyte-biased genes. (ZIP 2109 kb) [file 13059_2019_1630_MOESM3_ESM.zip › Con_GA_alignments/Ec-26_006560.aa_ali.fasta-gb.htm]

Ec-26\_006560.aa.fasta.trans.aa\_ali.fasta


## Gblocks 0.91b Results

Processed file: **Ec-26\_006560.aa.fasta.trans.aa\_ali.fasta**  
Number of sequences: **2**  
Alignment assumed to be: **Protein**  
New number of positions: **149** (selected positions are underlined in blue)

```
                         10        20        30        40        50        60
                 =========+=========+=========+=========+=========+=========+
Ec-26_006560     MDRTRSAAPAASWDTRAFVGDTTMCRPHSLSACGQQLSIPSQEGSKGLLTIGQRQQQQQE
MSTRG.30375.1    ------------------------------------------------------------
                                                                             


                         70        80        90       100       110       120
                 =========+=========+=========+=========+=========+=========+
Ec-26_006560     EGMAAPAATGQQAHRPPPRYTIRPPSRSFDGKFTLRQCSPPSVAELHANVVSSSSNFEFS
MSTRG.30375.1    ------------QQQSPPRFTVRPPSRSFDGRFTLRQCSPP-VPDWHGNVVTSSSNFDFS
                                 ######################### ##################


                        130       140       150       160       170       180
                 =========+=========+=========+=========+=========+=========+
Ec-26_006560     SSSNSAGGGGGGGARGLAACSGSDSDASMDAEVASLTSGRRLGSGAEGLVRMVNAPTKLP
MSTRG.30375.1    SSSNSGVARGEGGQ---QCCSGSDSDASTDAEVASLTNGRRLGTGAEGLLRMVSAPPRTR
                 #############      #########################################


                        190       200       210       220       230       240
                 =========+=========+=========+=========+=========+=========+
Ec-26_006560     RAEPRIRDAESAVALFALLSPSTHITPSPVQGRLQQQTTKQLDHLGDCGRSSWDTETGAG
MSTRG.30375.1    RGEPRIRDAESAVALFALLSPSARVTSSPTQDDLQKMHSGS----GDGRGSSWGTEASEA
                 ###################################          ###############


                        250       260       270
                 =========+=========+=========+====
Ec-26_006560     GEKGEEIWVDEPEAKRQRVASPSRCQTPKFNTHT
MSTRG.30375.1    DEEH----QGGGRAKRRRIAELVGPAVSRVPPA-
                 ##
```

```
Parameters used
Minimum Number Of Sequences For A Conserved Position: 2
Minimum Number Of Sequences For A Flanking Position: 2
Maximum Number Of Contiguous Nonconserved Positions: 8
Minimum Length Of A Block: 10
Allowed Gap Positions: None
Use Similarity Matrices: Yes
```

```
Flank positions of the 4 selected block(s)
Flanks: [77  101]  [103  133]  [140  215]  [226  242]  

New number of positions in Ec-26_006560.aa.fasta.trans.aa_ali.fasta-gb:  149  (54% of the original 274 positions)
```
